# Enhancing Nitrogen Nutrition Index estimation in rice using multi-leaf SPAD values and machine learning approaches

**DOI:** 10.3389/fpls.2024.1492528

**Published:** 2024-12-10

**Authors:** Yuan Wang, Peihua Shi, Yinfei Qian, Gui Chen, Jiang Xie, Xianjiao Guan, Weiming Shi, Haitao Xiang

**Affiliations:** ^1^ State Key Laboratory of Soil and Sustainable Agriculture, Changshu National Agro-Ecosystem Observation and Research Station, Institute of Soil Science, Chinese Academy of Sciences, Nanjing, China; ^2^ Department of Agronomy and Horticulture, Jiangsu Vocational College of Agriculture and Forestry, Jurong, China; ^3^ Soil and Fertilizer & Resources and Environmental Institute, Jiangxi Academy of Agricultural Sciences, Nanchang, China; ^4^ Institute of Biotechnology, Jiaxing Academy of Agricultural Science, Jiaxing, China

**Keywords:** rice nitrogen diagnosis, multi-leaf SPAD values, machine learning, leaf nitrogen concentration, nitrogen nutrition index, statistical metrics

## Abstract

Accurate nitrogen diagnosis is essential for optimizing rice yield and sustainability. This study investigates the potential of using multi-leaf SPAD measurements combined with machine learning models to improve nitrogen nutrition diagnostics in rice. Conducted across five locations with 15 rice cultivars, SPAD values from the first to fifth fully expanded leaves were collected at key growth stages. The study demonstrates that integrating multi-leaf SPAD data with advanced machine learning models, particularly Random Forest and Extreme Gradient Boosting, significantly improves the accuracy of Leaf Nitrogen Concentration (LNC) and Nitrogen Nutrition Index (NNI) estimation. The second fully expanded Leaf From the Top (2LFT) emerged as the most critical variable for predicting LNC, while the 3LFT was pivotal for NNI estimation. The inclusion of statistical metrics, such as maximum and median SPAD values, further enhanced model performance, underscoring the importance of considering both original SPAD measurements and derived indices. This approach provides a more precise method for nitrogen assessment, facilitating improved nitrogen use efficiency and contributing to sustainable agricultural practices through targeted and effective nitrogen management strategies in rice cultivation.

## 1 Introduction

Nitrogen is one of the most essential nutrients in crop growth, particularly in rice cultivation, where it plays a key role in photosynthesis, biomass accumulation, and overall yield (Zhang et al., 2021). As a staple food for over half of the world’s population, rice production is vital for global food security (Bandumula, 2018). Effective nitrogen management is crucial not only for maximizing rice yields but also for reducing environmental impacts associated with nitrogen over-application (Anas et al., 2020), such as water pollution and greenhouse gas emissions (Yu et al., 2019). Therefore, accurately understanding and monitoring the nitrogen status of rice plants is essential for optimizing nitrogen use efficiency and promoting sustainable agricultural practices (Colaço and Bramley, 2018; Berger et al., 2020).

Leaf Nitrogen Concentration (LNC) and Nitrogen Nutrition Index (NNI) are key indicators of a plant’s nitrogen status (Makowski et al., 2020; Li et al., 2022a). LNC represents the nitrogen content in the most important photosynthetic and assimilation organs, while NNI provides a relative measure of the nitrogen supply in relation to the plant’s needs. Accurate and timely estimation of these indices is crucial for effective nitrogen management, enabling farmers to apply the right amount of nitrogen at the right time to optimize crop performance (Yao et al., 2023).

The SPAD meter is a widely used tool for diagnosing crop nitrogen nutrition (Li et al., 2022b; Lu et al., 2022; Karaca et al., 2023; Wang et al., 2023; Wu et al., 2024). It measures chlorophyll content in leaves by comparing light absorption at specific wavelengths, which correlates with nitrogen levels. SPAD meters have achieved considerable success in diagnosing nitrogen nutrition in rice (Yuan et al., 2016b, 2016a; Ravier et al., 2017; Yang et al., 2018; Wu et al., 2024). However, these studies have often reported that diagnostic accuracy can be influenced by factors such as growth stage, cultivar, and environmental conditions (Ravier et al., 2017; Li et al., 2022b; Fu et al., 2023; Wang et al., 2023; Yao et al., 2023), leading to variability in results. Additionally, chlorophyll is only one form of nitrogen within the leaf, and a saturation effect on chlorophyll content can occur in specific leaves (Jiang et al., 2021). As a result, when chlorophyll content is high, the SPAD value is prone to being affected by this saturation effect (Gabriel et al., 2017; Souza et al., 2019).

To address these challenges, researchers have explored various methods. One approach involves using Relative SPAD (RSPAD) values to estimate nitrogen nutrition status, thereby minimizing the effects of cultivar and growth stage differences (Yue et al., 2020; Lu et al., 2022). Studies have shown that nitrogen-split application based on RSPAD can save nitrogen fertilizer and greatly improve nitrogen use efficiency (Wu et al., 2024). However, this method requires a well-fertilized reference area as a control, complicating practical applications and conflicting with the goal of simplifying nitrogen diagnostics (Ravier et al., 2017; Yue et al., 2020; Lu et al., 2022). Another approach uses SPAD indices, such as differences and ratios between SPAD values of different leaf positions, to enhance diagnostic accuracy (Zhang et al., 2019; Karaca et al., 2023). While this method shows promise, it lacks validation on large-scale datasets, limiting its applicability.

Machine learning methods offer significant advantages in overcoming these limitations by effectively handling complex nonlinear relationships in agricultural data (Chlingaryan et al., 2018). By integrating multi-leaf SPAD measurements with machine learning algorithms, it is possible to enhance the accuracy of nitrogen nutrition diagnosis in rice. This integration allows for better interpretation of SPAD data affected by factors like growth stage and cultivar, thus improving nitrogen management strategies (Yang et al., 2023; Mandal et al., 2024).

Rice leaves differ significantly in their physiological characteristics depending on their position on the plant (Sun et al., 2018). These differences can affect the accuracy of nitrogen nutrition diagnosis, as leaves at various positions respond differently to nitrogen availability (Yuan et al., 2016b; Zhao et al., 2018). For instance, younger leaves at the top of the plant are in dynamic growth phases with higher nitrogen content, while older leaves are more stable or in senescence, affecting their SPAD readings (Zhang et al., 2019; Li et al., 2022b; Wang et al., 2023). By leveraging information from multiple leaf positions and exploring the relationships between SPAD values at these positions and the plant’s nitrogen status, the accuracy of nitrogen nutrition diagnosis in rice can be improved (Yuan et al., 2016a).

In this study, we conducted multi-location, multi-cultivar field trials in rice, collecting data across various growth stages to build a comprehensive dataset. We applied several machine learning methods and performed feature importance analyses to evaluate the role of multi-leaf SPAD information in enhancing nitrogen nutrition diagnosis. This research not only contributes to improving the accuracy of nitrogen diagnostics in rice but also offers insights into the future prospects of integrating machine learning with agronomic practices.

## 2 Materials and methods

### 2.1 Study area and experimental design

**Figure 1:**
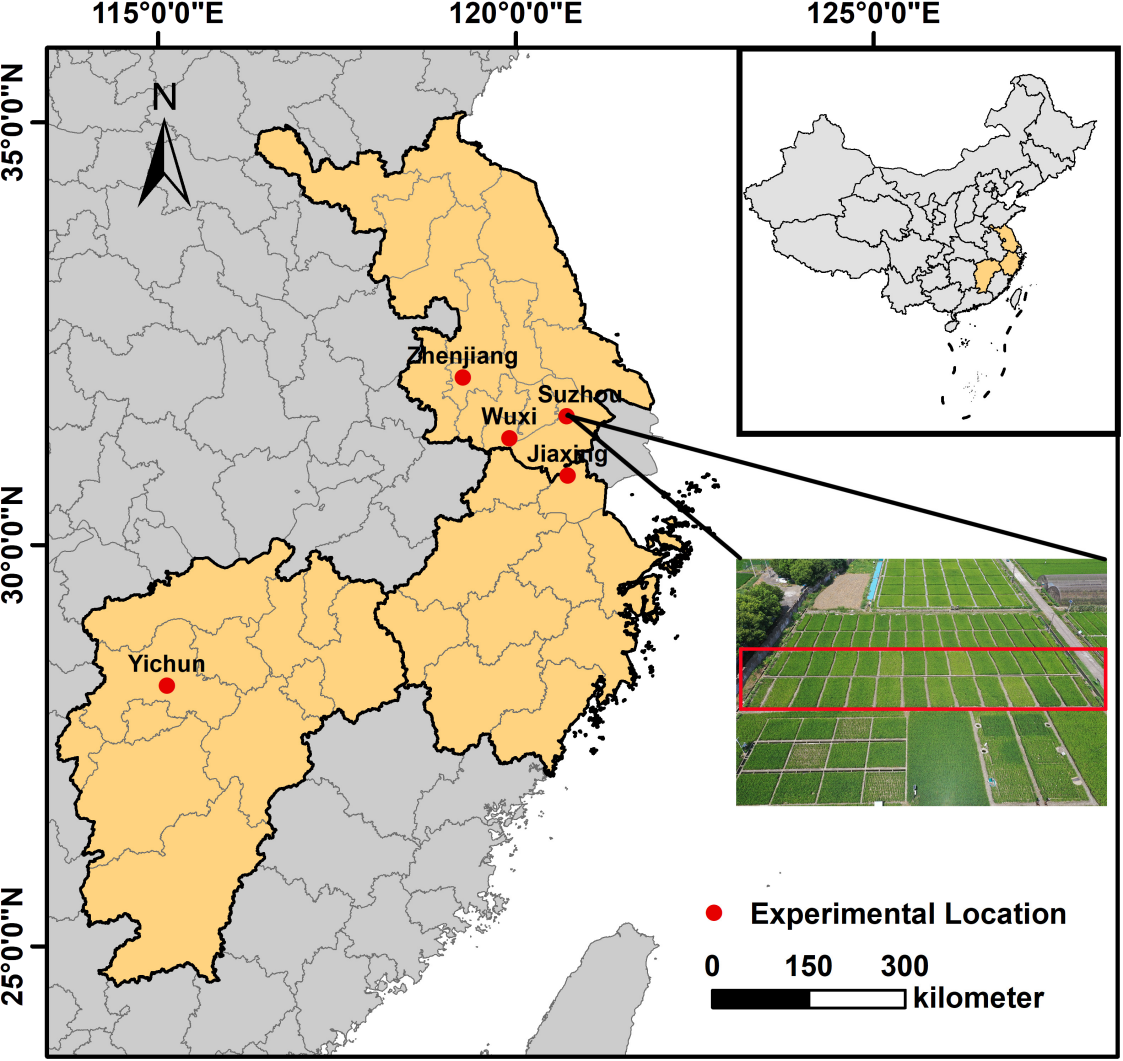
Geographic locations of the five experimental sites in Jiangsu, Zhejiang, and Jiangxi provinces, China, along with representative image of field experiments.

This study was conducted across five locations within the Jiangsu, Zhejiang, and Jiangxi provinces, situated in the middle and lower reaches of the Yangtze River in China: Suzhou, Wuxi, Zhenjiang, Jiaxing, and Yichun prefecture-level cities (**Figure 1**). Seventeen field experiments were conducted, involving fifteen rice cultivars—five indica and ten japonica cultivars. The field experiments were arranged in a randomized block design, with each experiment comprising two to seven nitrogen fertilizer treatments, including at least a control with no nitrogen and a conventional nitrogen application to induce varied nitrogen statuses in the rice plants. Each treatment was replicated across three to four plots, with plot sizes ranging from 30 to 100 m². Detailed information on each experiment and experimental site, including soil types, is provided in **Tables 1** and **2**. All field management practices, except nitrogen levels, conformed to local conventional cultivation methods.

**Table 1:** Summary of field experiment information.

No.	Cultivars	Subspecies	Rice Type	Number of Nitrogen Treatments	Experimental Site
1	NG46_1	japonica	Conventional	6	Suzhou
2	NG46_2	japonica	Conventional	6	Suzhou
3	NG46_3	japonica	Conventional	6	Wuxi
4	WYG35	japonica	Conventional	6	Wuxi
5	NG5055	japonica	Conventional	7	Wuxi
6	J67	japonica	Conventional	3	Jiaxing
7	XS14	japonica	Conventional	6	Jiaxing
8	JH218	japonica	Conventional	5	Jiaxing
9	YG13	japonica	Conventional	5	Zhenjiang
10	CY5	japonica	hybrid	4	Yichun
11	CY6	japonica	hybrid	5	Suzhou
12	JYZK6	japonica	hybrid	2	Jiaxing
13	HHZ	indica	Conventional	4	Yichun
14	MXXZ	indica	Conventional	4	Yichun
15	TY398	indica	hybrid	4	Yichun
16	TYXZ	indica	hybrid	4	Yichun
17	TYHZ	indica	hybrid	4	Yichun

Each treatment has 3-4 replicates.

**Table 2:** Climatic and soil conditions for each experimental site.

Experimental Site	Average annual temperature (°C)	Annual precipitation (mm)	sunshine hours (h)	Soil Type
Suzhou	16.9	1615	1711	Gleyi-Stagnic Anthrosols
Wuxi	17.1	1553	1898	Hapli-Stagnic Anthrosols
Jiaxing	15.9	1168	2017	Hapli-Stagnic Anthrosols
Zhenjiang	15.1	1019	2116	Hapli-Stagnic Anthrosols
Yichun	17.5	1560	1780	Argi-Udic Ferrosols

Soil types are classified according to the Chinese Soil Taxonomy (CST).

### 2.2 Data collection and plant sample testing

Samples were collected at regular intervals of 10-15 days throughout the rice growing season, covering the main growth stages: Tillering (TI), Stem Elongation (SE), Panicle Initiation (PI), Heading (HD), and Grain Filling (GF) (the GF stage sampling was only for the varieties NG46_1, WYG35, and TY398). Due to varietal differences and planting locations, different rice cultivars may be at different growth stages on the same sampling date. To account for this, we scheduled destructive sampling at fixed intervals and, during data analysis, grouped the samples according to the actual growth stages of each cultivar.

At each stage, three to six uniformly growing plants were destructively sampled from the experimental plots. The plants were divided into leaves, sheaths, and panicles, which were oven-dried at 105°C for 30 minutes, followed by further drying at 75°C to a constant weight. The dried samples from each organ were weighed, ground, and the nitrogen concentration was determined using the Kjeldahl method. The overall plant nitrogen concentration was calculated as the ratio of total nitrogen content to the total dry mass.

The NNI was calculated as the ratio of plant nitrogen concentration to the critical nitrogen concentration (N_critical_). An NNI close to 1 indicates optimal nitrogen supply, while an NNI greater than 1 suggests excess nitrogen (where additional nitrogen does not increase biomass). An NNI less than 1 indicates nitrogen deficiency, with the severity of deficiency inversely related to the NNI value. The method for determining the N_critical_ in this study was based on the approach of Justes et al. (1994). For each experiment and sampling date, variance analysis of dry matter (DM) was conducted across treatments, followed by LSD testing to identify the treatment with the highest biomass but the lowest plant nitrogen concentration, designated as the N_critical_. To reduce the likelihood of Type II errors in the LSD test, the significance level was set at 0.10. For experiments with only two nitrogen levels or where the N_critical_ could not be determined using this method, a Critical Nitrogen Dilution Curve (CNDC) was plotted using other confirmed N_critical_, and the NNI was calculated from this curve. The CNDC established in this study was ${\text{N}}_{\text{critical}}=3.44{\text{DM}}^{-0.44}$ .

Simultaneously with the destructive sampling, 15 to 20 rice plants were selected from each experimental plot to measure the SPAD values of the first to fifth fully expanded Leaves From the Top (1-5 LFT). Each leaf was measured at three positions (middle, upper third, and lower third) on one side of the main vein, and the SPAD values were averaged. SPAD measurements were performed using a SPAD-502 PLUS (Konica Minolta) chlorophyll meter.

### 2.3 Estimation of rice LNC and NNI

This study employed several modeling techniques to predict LNC and the NNI in rice, including univariate Linear Regression (LR), Partial Least Squares (PLS) regression, Support Vector Regression (SVR), Random Forest (RF), and Extreme Gradient Boosting (XGB).

PLS regression addresses multicollinearity by projecting predictors and responses onto latent variables that maximize their covariance. The key hyperparameter in PLS is the number of latent components, which strikes a balance between dimensionality reduction and predictive accuracy. SVR aims to find the optimal hyperplane for data fitting, with critical hyperparameters including the regularization parameter *C*, the kernel function [e.g., linear, polynomial, or Radial Basis Function (RBF)], the kernel coefficient *γ* for RBF, and the epsilon (*ϵ*) margin. RF builds a forest of decision trees, with hyperparameters such as the number of trees, maximum tree depth, minimum samples required for node splitting, and the maximum number of features considered at each split, controlling model complexity and diversity. XGB constructs sequential trees to correct errors, with essential hyperparameters including learning rate, number of boosting iterations, tree depth, subsample ratios for training instances and features, and L1/L2 regularization to mitigate overfitting. These models were chosen for their ability to capture complex, nonlinear relationships between SPAD measurements and nitrogen status indicators. Machine learning methods like RF and XGB are particularly effective in handling high-dimensional data and interactions among variables, which are common in agronomic datasets.

Hyperparameter tuning was conducted using Bayesian optimization through the ‘Optuna’ framework (Akiba et al., 2019), combined with five-fold cross-validation in Python 3.12.0. The hyperparameters tested are detailed in **Table 3**.

**Table 3:** Hyperparameters and tested range for each modeling method.

Modeling Method	Hyperparameter	Tested Range
PLS	Number of components	[1, 5], Integer
SVR	Regularization parameter (*C*)	[10^-3^, 10^3^], Log-uniform (float)
	Kernel coefficient (*γ*)	[10^-3^, 10], Log-uniform (float)
	Epsilon (*ϵ*)	[10^-4^, 1], Log-uniform (float)
RF	Number of trees	[50, 500], Integer
	Tree depth	[1, 20], Integer
	Minimum samples to split	[2, 11], Integer
	Minimum samples per leaf	[1, 11], Integer
	Maximum features for split	sqrt, log2, None
XGB	Learning rate	[10^-3^, 10^-1^], Log-uniform (float)
	Boosting iterations	[50, 500], Integer
	Tree depth	[3, 10], Integer
	Subsample ratio (instances)	[0.5, 1.0], Uniform (float)
	Subsample ratio (features)	[0.5, 1.0], Uniform (float)
	L1 regularization term (α)	[0.1, 1.0], Uniform (float)
	L2 regularization term (λ)	[0.1, 1.0], Uniform (float)

Values within square brackets indicate the hyperparameter search range. “Integer” denotes that the hyperparameter values are integers sampled within the specified range. “Uniform (float)” indicates that the hyperparameter values are floating-point numbers sampled uniformly from the specified range, while “Log-uniform (float)” indicates that the values are sampled from a logarithmic distribution within the specified range.

In the LR model, the SPAD value from a single leaf position was used as the input to estimate rice LNC and NNI. For the other models, which accommodate multiple input variables, the variables were grouped based on original SPAD measurements, SPAD indices between different leaf positions, and SPAD statistical metrics. Detailed information on the input variables for each group is provided in **Table 4**.

**Table 4:** Input variable combinations for the rice nitrogen estimation model.

Variable Combination	Description	Included Variables
comb_1	SPAD values of the five fully expanded leaves from the top of rice plant	1LFT, 2LFT, 3LFT, 4LFT, 5LFT
comb_2	Normalized difference index, ratios, and difference of SPAD values between specific leaf positions	ND2_1, ND3_1, ND3_2, ND4_1, ND4_2, ND4_3, R2_1, R3_1, R3_2, R4_1, R4_2, R4_3, D2_1, D3_1, D3_2, D4_1, D4_2, D4_3
comb_3	Combination of comb_2 variables with additional statistical metrics (std, min, max, median) of SPAD values	ND2_1, ND3_1, ND3_2, ND4_1, ND4_2, ND4_3, R2_1, R3_1, R3_2, R4_1, R4_2, R4_3, D2_1, D3_1, D3_2, D4_1, D4_2, D4_3, 1LFT_std, 1LFT_min, 1LFT_max, 1LFT_median, 2LFT_std, 2LFT_min, 2LFT_max, 2LFT_median, 3LFT_std, 3LFT_min, 3LFT_max, 3LFT_median
comb_4	Combination of all the above variables including original SPAD measurements, SPAD indices, and statistical metrics	All the above variables

1-5LFT refers to the 1st, 2nd, 3rd, 4th and 5th fully expanded leaf form the top of the rice plant, where SPAD measurements are taken. The variables NDi_j, Ri_j, and Di_j represent the Normalized Difference Index, Ratio Index, and Difference between SPAD values at iLFT and jLFT, respectively, calculated as (SPAD_iLFT_−SPAD_jLFT_)/(SPAD_iLFT_+SPAD_jLFT_), SPAD_iLFT_/SPAD_jLFT_, and SPAD_iLFT_−SPAD_jLFT_. The variables std, min, max, and median represent the standard deviation, minimum, maximum, and median of the SPAD values from the corresponding leaves.

### 2.4 Model evaluation and feature importance analysis

The performance of each model was evaluated using the coefficient of determination (R²), Root Mean Square Error (RMSE), and Average Testing Prediction Accuracy (ATPA). These indicators were calculated for both the training and validation datasets, which were split in an 8:2 ratio, to assess the models’ ability to generalize to unseen data. The ATPA was calculated using the following formula:


$$\text{ATPA}=\left(1-\frac{1}{n}\sum_{i=1}^{n}\frac{\left|TA_{i}-TP_{i}\right|}{TA_{i}}\right)\times 100$$

where *TA_i_* represents the observed value, *TP_i_* represents the predicted value, and *n* is the number of observations.

Feature importance was analyzed using SHapley Additive exPlanations (SHAP) to determine the contribution of each input variable to the model’s predictions (Lundberg and Lee, 2017). SHAP values provided insights into the influence of each feature on prediction outcomes, enabling a detailed understanding of the models’ decision-making processes. This analysis offered both global interpretation—by averaging SHAP values across the entire dataset to assess overall feature importance—and local interpretation, by examining individual data points to understand specific feature contributions. In this study, SHAP analysis was conducted for the RF and XGB models using the four input variable combinations (**Table 4**).

## 3 Results

### 3.1 Distribution of SPAD values across different leaf positions in rice

**Figure 2:**
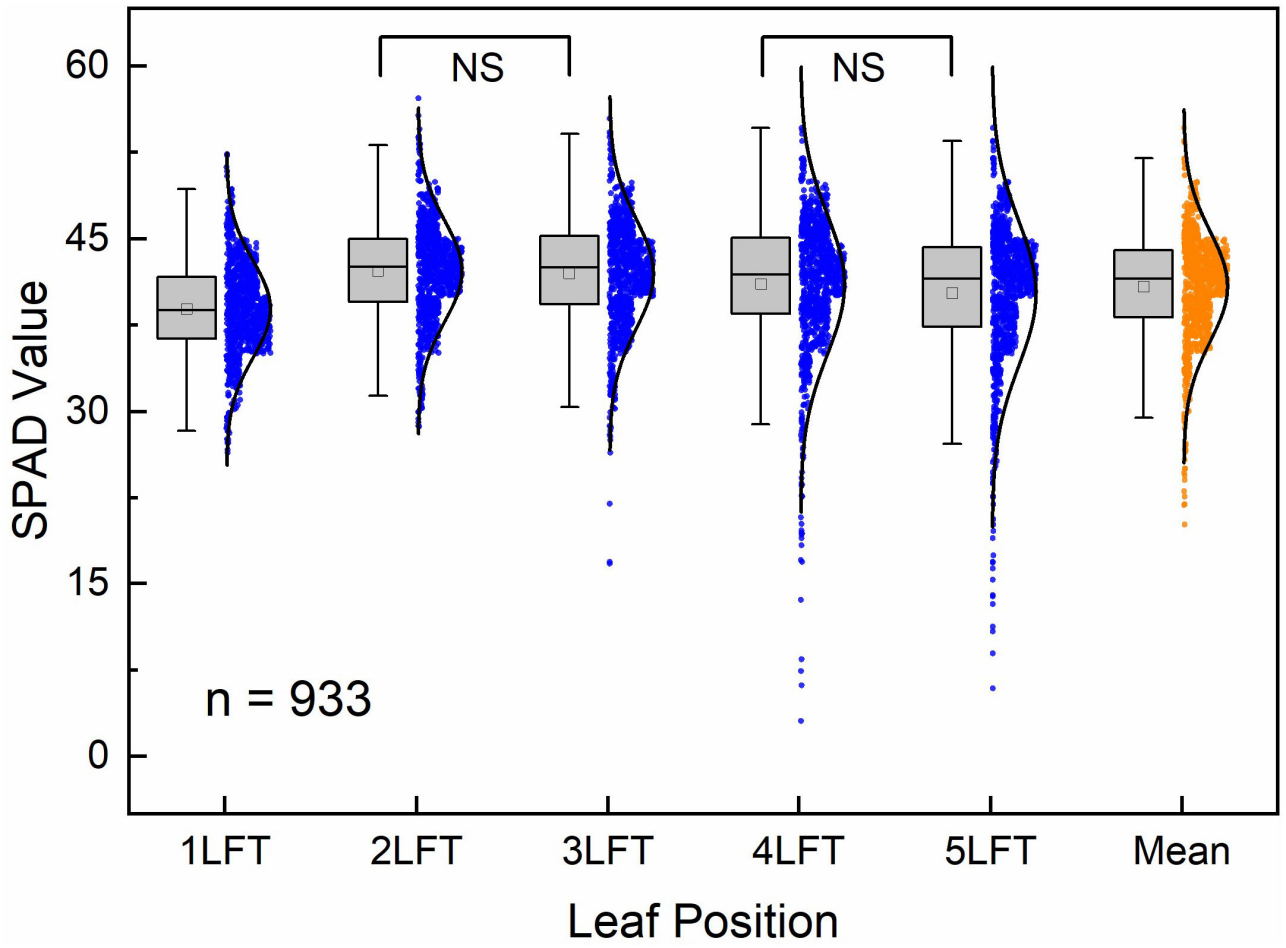
Distribution of SPAD values across different leaf positions and their means in all rice cultivars. Each box plot displays the range of SPAD values, with whiskers extending to 1.5 times the interquartile range, a horizontal line representing the median, and a point within the box indicating the mean. Accompanying density distribution curves and scatter plots provide additional insights into the data spread and distribution shape. Significant differences in SPAD values are present among all leaf positions, except those marked as “NS”.

A statistical analysis of SPAD values across different leaf positions in all rice cultivars revealed significant differences in the distribution of SPAD values from the 1LFT to 5LFT (**Figure 2**). The SPAD values from the 1LFT to 5LFT followed a trend of initially increasing, and then gradually decreasing. The 1LFT recorded the lowest average SPAD value of 38.9, whereas the highest average SPAD value of 42.2 was observed in the 2LFT. After the peak, the average SPAD values diminished progressively, with an accompanying increase in the range of SPAD value distributions, indicating greater variability in older leaves.

Variance analysis revealed no significant differences in average SPAD values between 2LFT and 3LFT or between 4LFT and 5LFT, while significant differences were detected between the other leaf positions (p < 0.05). As the leaves aged from 1LFT to 5LFT, the difference between the median and mean SPAD values increased, with values of -0.09, 0.36, 0.55, 0.84, and 1.26, respectively. This suggests a growing skew in SPAD values with leaf age, where older leaves tend to have lower SPAD values, causing the mean to decline relative to the median. The statistical analysis of SPAD values at different leaf positions indicates that specific leaf positions exhibit distinct responses to changes in nitrogen concentration and growth stages. This finding suggests that selecting appropriate leaf positions for SPAD measurements is crucial for accurate nitrogen monitoring in rice.

### 3.2 Simple Linear Regression for LNC and NNI estimation

A simple Linear Regression (LR) analysis was conducted using SPAD values from individual leaf positions (1LFT to 5LFT) and their average to estimate rice LNC and NNI. The overall mean LNC across all samples was 2.96%, with lower and upper quartiles of 2.42% and 3.43%. The mean NNI was 0.94, with quartiles of 0.74 and 1.12.

Across 17 independent experiments, SPAD values effectively estimated LNC and NNI for most cultivars, with R² values ranging from 0.14 to 0.92 (**Tables 5**, **6**). Cultivars NG5055, J67, and MXXZ exhibited the highest estimation performance, while WYG35, XS14, and TY398 showed lower R² values for LNC estimation. Similarly, for NNI estimation, WYG35, XS14, and JYZK6 had lower R² values. No significant difference in estimation performance was found between japonica and indica subspecies.

**Table 5:** Linear regression analysis between SPAD values and LNC (%) at different leaf positions.

Cultivars	1LFT		2LFT		3LFT		4LFT		5LFT		Average SPAD	
	R^2^	RMSE	R^2^	RMSE	R^2^	RMSE	R^2^	RMSE	R^2^	RMSE	R^2^	RMSE
NG46_1	0.48(1)	0.27	0.67	0.22	0.76	0.19	0.77	0.19	0.79	0.18	0.79	0.18
NG46_2	0.63	0.24	0.73	0.19	0.73	0.20	0.64	0.21	0.71	0.20	0.74	0.19
NG46_3	0.61	0.21	0.73	0.19	0.74	0.18	0.65(1)	0.20	0.74	0.18	0.76	0.17
WYG35	0.19(2a)	0.28	0.25(1a)	0.27	0.39(1a)	0.23	0.50	0.21	0.45(a)	0.22	0.45(a)	0.22
NG5055	0.66	0.24	0.82	0.19	0.84	0.18	0.73	0.23	0.80	0.20	0.85	0.18
J67	0.67	0.20	0.82	0.14	0.79	0.16	0.80	0.16	0.71	0.18	0.82	0.14
XS14	0.38(1)	0.22	0.57(1)	0.18	0.58	0.18	0.42(1)	0.22	0.30(1)	0.24	0.51(1)	0.19
JH218	0.68	0.23	0.77	0.21	0.60	0.29	0.45(1)	0.34	0.55	0.30	0.68	0.26
YG13	0.56(a)	0.16	0.64(a)	0.14	0.65	0.15	0.43(1)	0.19	0.56	0.17	0.62	0.15
CY5	0.60(1)	0.18	0.62(1)	0.17	0.55(1)	0.19	0.51(1a)	0.20	0.63(1)	0.19	0.61(1)	0.18
CY6	0.64	0.22	0.86	0.14	0.82	0.16	0.59(1)	0.24	0.77	0.18	0.81	0.17
JYZK6	0.82	0.22	0.88	0.17	0.92	0.14	0.86	0.19	0.90	0.16	0.90	0.16
HHZ	0.69	0.26	0.53	0.31	0.61	0.28	0.57	0.30	0.49(1)	0.33	0.60	0.29
MXXZ	0.85	0.18	0.82	0.22	0.86	0.19	0.58(1)	0.30	0.72	0.27	0.82	0.20
TY398	0.44(2)	0.32	0.48(1a)	0.31	0.47(1a)	0.31	0.46(2)	0.31	0.21(2a)	0.39	0.48(1a)	0.30
TYXZ	0.43(1)	0.34	0.67	0.25	0.78	0.21	0.70	0.23	0.79	0.20	0.75	0.22
TYHZ	0.63	0.24	0.70	0.22	0.77	0.19	0.71	0.21	0.63	0.24	0.76	0.19
Mean	0.59	0.24	0.68	0.21	0.70	0.20	0.61	0.23	0.63	0.23	0.70	0.20

The R² and RMSE values for each cultivar represent the averages of regression analyses conducted separately across different growth stages. Numbers in parentheses indicate the number of growth stages where the regression coefficients for that cultivar are not statistically significant. The letter ‘a’ indicates that the regression coefficients for one growth stage are significant at the 0.1 level. All other unmarked regression models have coefficients that are significant at the 0.05 level.

**Table 6:** Linear regression analysis between SPAD values and NNI at different leaf positions.

Cultivars	1LFT		2LFT		3LFT		4LFT		5LFT		Average SPAD	
	R^2^	RMSE	R^2^	RMSE	R^2^	RMSE	R^2^	RMSE	R^2^	RMSE	R^2^	RMSE
NG46_1	0.45(1)	0.18	0.66	0.14	0.77	0.12	0.74	0.13	0.77	0.12	0.77	0.12
NG46_2	0.68	0.11	0.79	0.08	0.79	0.09	0.74	0.09	0.80	0.09	0.83	0.08
NG46_3	0.57	0.11	0.61(a)	0.10	0.65(a)	0.09	0.61(1)	0.10	0.67	0.09	0.68	0.09
WYG35	0.29(1)	0.13	0.29(a)	0.13	0.35(a)	0.13	0.38(a)	0.12	0.43	0.12	0.43	0.12
NG5055	0.68	0.11	0.80	0.09	0.81	0.08	0.74	0.09	0.78	0.09	0.84	0.08
J67	0.76	0.09	0.90	0.06	0.88	0.06	0.85	0.07	0.79	0.08	0.91	0.06
XS14	0.31(1)	0.13	0.56(a)	0.10	0.58	0.10	0.37(1)	0.12	0.27(1)	0.14	0.47(1)	0.11
JH218	0.46	0.12	0.64	0.10	0.81	0.07	0.68	0.09	0.81	0.07	0.81	0.07
YG13	0.47	0.06	0.69	0.05	0.67	0.05	0.53(1)	0.05	0.67	0.05	0.68	0.05
CY5	0.54(1)	0.06	0.57(1)	0.06	0.61(1)	0.06	0.60(1)	0.06	0.71(a)	0.06	0.64(1)	0.05
CY6	0.48	0.14	0.78	0.10	0.80	0.09	0.63	0.12	0.77	0.10	0.79	0.09
JYZK6	0.56(1a)	0.19	0.54(1)	0.18	0.52(1)	0.19	0.52(1a)	0.19	0.56(1)	0.18	0.54(1)	0.19
HHZ	0.62	0.13	0.60	0.13	0.60	0.13	0.62	0.13	0.50(a)	0.15	0.62	0.13
MXXZ	0.77	0.10	0.80	0.09	0.87	0.07	0.61(a)	0.13	0.63	0.13	0.79	0.10
TY398	0.59(b)	0.10	0.67(a)	0.09	0.66	0.09	0.68	0.09	0.39(1)	0.14	0.68	0.09
TYXZ	0.60	0.13	0.78	0.10	0.91	0.06	0.83	0.09	0.90	0.07	0.90	0.07
TYHZ	0.59	0.10	0.59(a)	0.10	0.64(a)	0.09	0.65	0.09	0.37(1)	0.13	0.61	0.10
Mean	0.55	0.12	0.66	0.10	0.70	0.09	0.63	0.10	0.64	0.11	0.70	0.09

The R² and RMSE values for each cultivar represent the averages of regression analyses conducted separately across different growth stages. Numbers in parentheses indicate the number of growth stages where the regression coefficients for that cultivar are not statistically significant. The letters ‘a’ or ‘b’ indicate that the regression coefficients for one or two growth stages, respectively, are significant at the 0.1 level. All other unmarked regression models have coefficients that are significant at the 0.05 level.

The estimation accuracy varied with leaf position. The average R² values increased from 1LFT, peaked at 3LFT, and then decreased, with 3LFT providing the most accurate estimates for both LNC and NNI (average R² of 0.70). In contrast, 1LFT had the lowest estimation accuracy.

**Figure 3:**
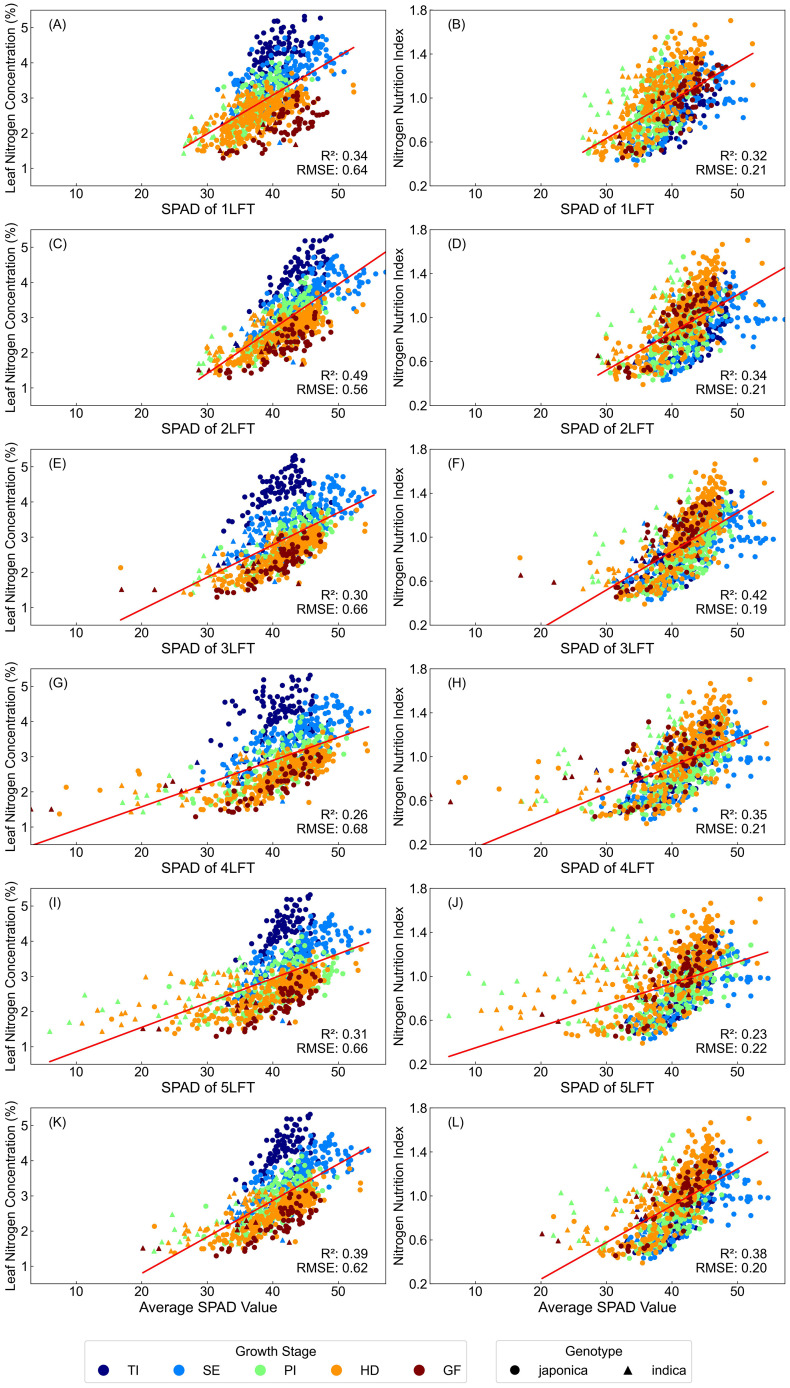
Linear regression analysis between SPAD readings from different leaf positions and LNC and NNI across different growth stages and cultivars (n=933). **(A–J)** represent scatter plots for SPAD readings from the 1st to 5th leaf from the top (LFT) and their corresponding LNC **(A, C, E, G, I)** or NNI **(B, D, F, H, J)** values. **(K, L)** show the scatter plots for the average SPAD readings across the 1st to 5th LFT and their corresponding LNC **(K)** or NNI **(L)** values.

When LR was applied to the pooled dataset including all cultivars and growth stages, R² values decreased to a maximum of 0.49 for LNC and 0.42 for NNI, indicating that variability among cultivars and growth stages significantly impacted regression accuracy (**Figure 3**). For LNC estimation, 2LFT provided the highest accuracy, while for NNI estimation, 3LFT was most accurate. Using the average SPAD values from 1-5LFT offered estimation accuracy close to that of the best individual leaf positions.

Scatter plots of LNC versus SPAD values (**Figure 3**) revealed that early growth stages had higher nitrogen concentrations, which declined as rice developed. However, SPAD values remained similar across growth stages, causing data grouping by growth stage and reducing LR model accuracy. For NNI versus SPAD plots, NNI values clustered between 0.5 and 1.5, mitigating the grouping effect, but LR accuracy did not improve compared to LNC estimation.

Regarding rice subspecies, indica clustered in the lower SPAD value range, while japonica was concentrated in the higher SPAD value range. This difference likely reflects variations in nitrogen uptake and utilization between the two subspecies.

### 3.3 PLS and machine learning for LNC and NNI estimation

**Figure 4:**
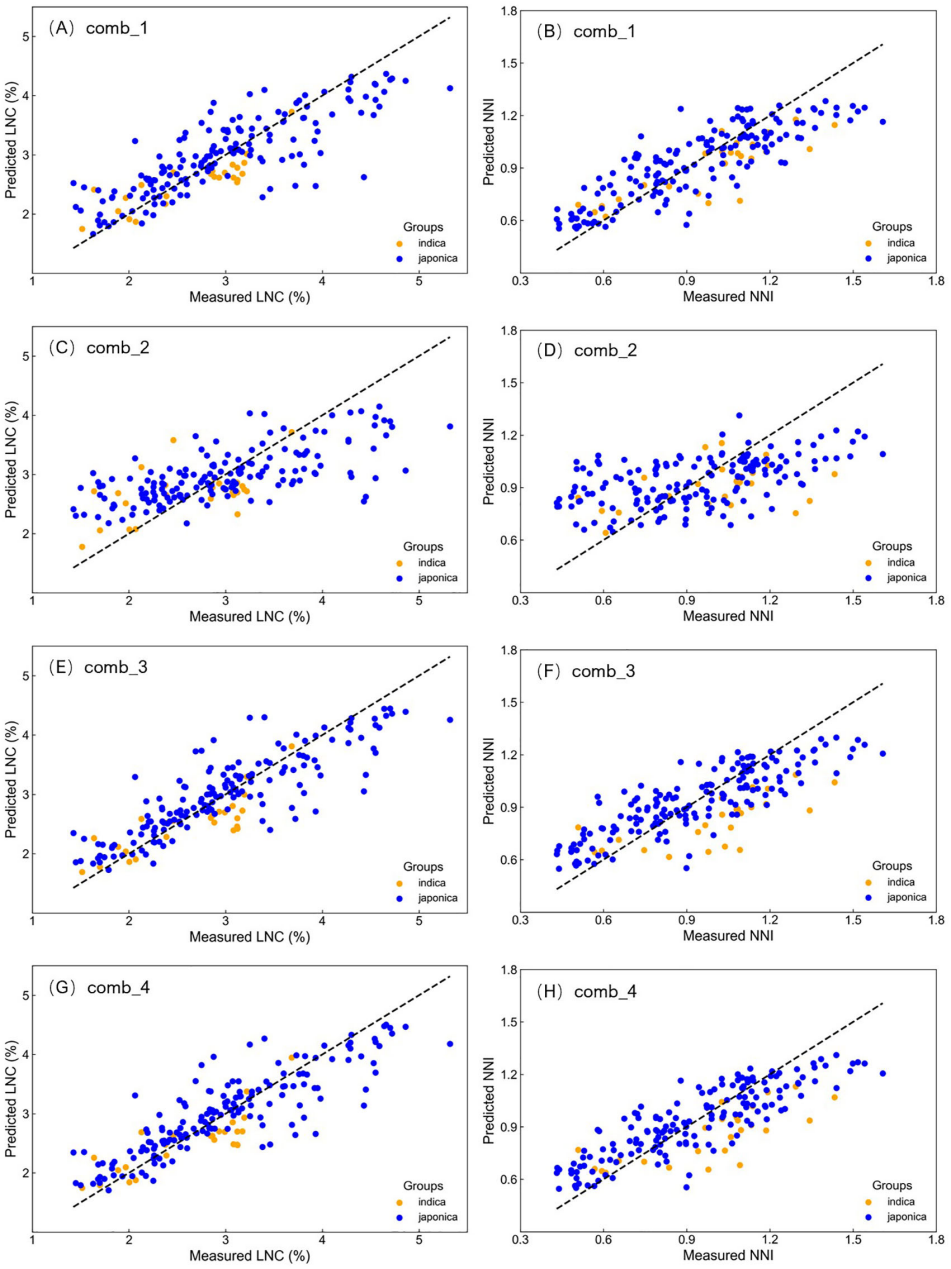
Validation results for predicted vs. measured LNC **(A, C, E, G)** and NNI **(B, D, F, H)** using random forest with input variable combinations comb_1, comb_2, comb_3, and comb_4, respectively. Each plot shows 1:1 scatter relationships.

Using the pooled dataset, four groups of feature variables—comprising leaf SPAD values, SPAD indices, and SPAD statistical metrics—were used as inputs for PLS regression, SVR, RF, and XGB models to estimate rice LNC and NNI. Compared to the results of LR using SPAD values from single leaf positions, models incorporating multiple feature variables significantly improved estimation accuracy, with R² values increasing from 0.3-0.4 to 0.5-0.7 (**Table 7**; **Figure 4**). Among the four modeling methods, SVR, RF, and XGB showed similar performance, with the RF model achieving the highest accuracy in estimating LNC. Notably, when comb_3 and comb_4 were used as input variables, the R² values reached 0.73 and 0.74, respectively, and the ATPA also achieved the highest values among all models, at 88.84 and 88.89, respectively. For NNI estimation, the RF and XGB models performed consistently, with average ATPA values of 83.44 and 83.61, respectively. While the SVR model showed slightly lower R² values compared to RF and XGB, its ATPA was comparable to the other two models. The PLS model, although effective, performed slightly worse than the machine learning models.

**Table 7:** Validation results of different modeling methods based on the pooled dataset.

Modeling Method	Input Variable	LNC Estimation			NNI Estimation		
		R^2^	RMSE(%)	ATPA	R^2^	RMSE	ATPA
PLS	comb_1	0.59	0.53	85.55	0.55	0.18	82.62
	comb_2	0.53	0.56	83.85	0.32	0.22	77.68
	comb_3	0.62	0.51	86.22	0.52	0.19	82.37
	comb_4	0.62	0.51	86.17	0.58	0.18	83.83
SVR	comb_1	0.71	0.44	88.58	0.68	0.15	86.18
	comb_2	0.51	0.57	82.66	0.33	0.22	78.29
	comb_3	0.69	0.46	88.16	0.58	0.18	84.09
	comb_4	0.69	0.46	88.24	0.66	0.16	85.90
RF	comb_1	0.68	0.46	87.60	0.69	0.15	86.23
	comb_2	0.44	0.61	82.13	0.34	0.22	78.03
	comb_3	0.73	0.42	88.84	0.62	0.17	84.21
	comb_4	0.74	0.42	88.89	0.67	0.16	85.30
XGB	comb_1	0.68	0.47	87.34	0.68	0.15	85.93
	comb_2	0.58	0.53	84.73	0.36	0.22	78.61
	comb_3	0.69	0.45	88.34	0.63	0.17	84.12
	comb_4	0.69	0.46	88.31	0.69	0.15	85.77

Model training and validation, using an 8:2 split, were conducted on a pooled dataset comprising data from all varieties and growth stages. The results shown in the table are from the model’s performance on the validation dataset.

Overall, the models provided better estimation accuracy for LNC than for NNI, with average ATPA values of 86.60 and 83.07, respectively. Among the four input variable combinations, using the second combination (comb_2)—which includes normalized difference indices, ratios, and differences of SPAD values—resulted in significantly lower estimation accuracy for both LNC and NNI compared to the other three combinations, particularly for NNI estimation. In contrast, combinations that included original SPAD values from specific leaf positions (comb_1, comb_4) and SPAD statistical metrics (comb_3, comb_4) provided higher estimation accuracy for both LNC and NNI. The accuracy of the models followed the trend comb_4 > comb_1 > comb_3, indicating that the inclusion of multi-leaf position SPAD measurements and more comprehensive variable combinations improved prediction accuracy.

Using the RF model as an example, a 1:1 plot of predicted versus observed values on the validation dataset was generated (**Figure 4**). The distribution of data points demonstrates that the combination of multi-leaf SPAD features with machine learning methods effectively eliminated the grouping effect observed in LR models when using data from different growth stages, significantly improving the model’s accuracy in estimating LNC and NNI. However, across all input variables and target variables, the models tended to underestimate in the high-value regions, particularly when using comb_2 as input. This underestimation may be attributed to the saturation effect of SPAD values in estimating nitrogen concentration and the nitrogen dilution effect observed as the plant grows.

### 3.4 Feature importance of the random forest model

**Figure 5:**
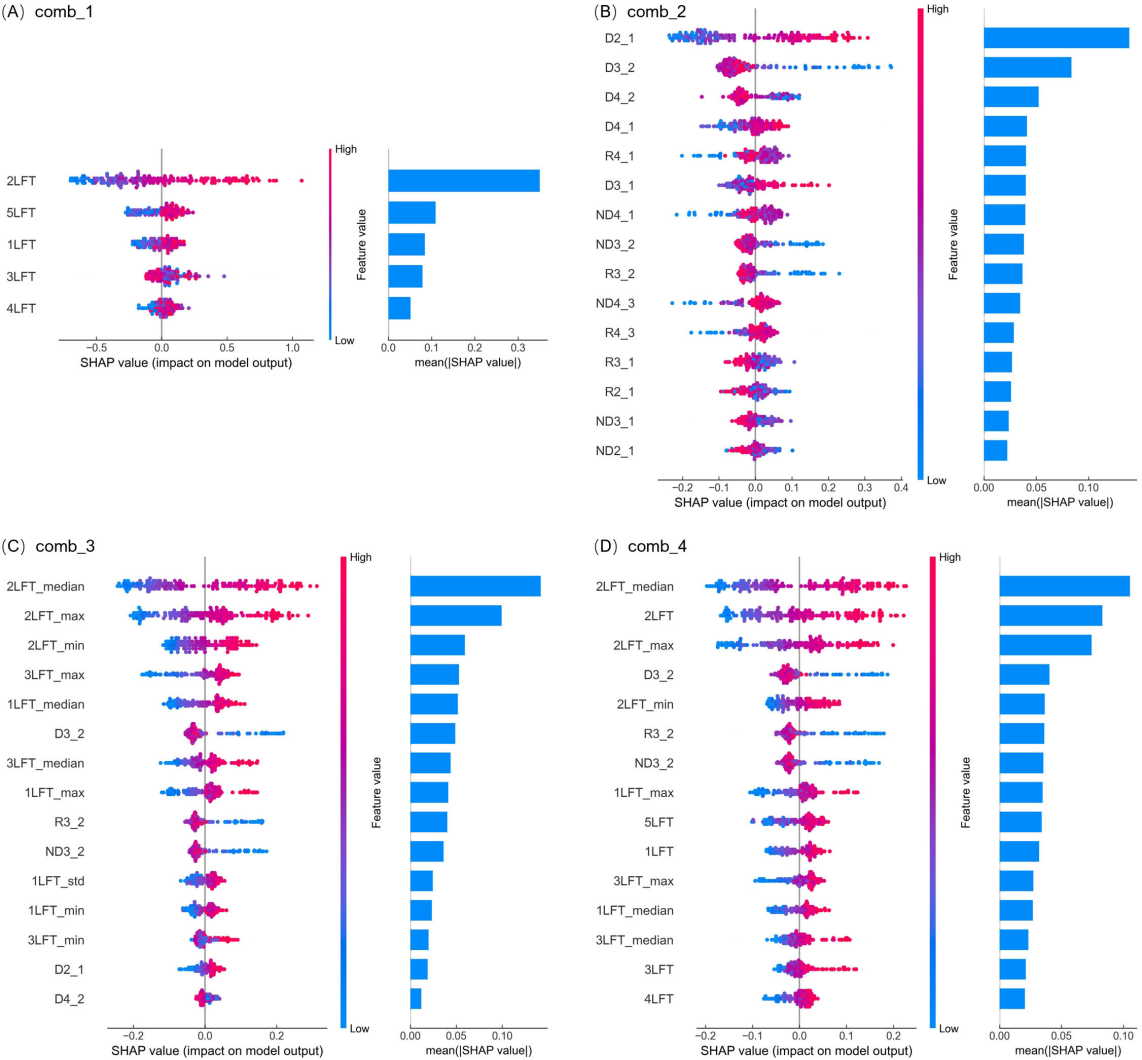
SHAP analysis of feature importance in predicting rice LNC with the random forest model across four input variable combinations. **(A–D)** correspond to the results for input variable combinations comb_1, comb_2, comb_3, and comb_4, respectively. The left side of each subplot shows SHAP value distributions, where each dot represents a data point from the validation dataset. The x-axis indicates the feature's impact on the model's prediction, and the dot color reflects the feature value, ranging from blue (low) to pink (high). Positive SHAP values indicate that the feature increases the prediction, while negative values indicate a decrease. The right side presents a bar chart (sharing the Y-axis with the left-side chart), with features ranked in descending order of their average absolute SHAP values, highlighting the most influential variables for the model's accuracy.

SHAP analysis was performed on the input features of the RF model to assess their impact on predicting LNC and NNI in rice. When only SPAD measurements from the 1-5LFT were used as input variables (**Figure 5A**), the 2LFT emerged as the most influential feature for LNC estimation, with an average SHAP value of approximately 0.35, significantly higher than those of the other leaf positions. The second most important feature was the 5LFT, with an average SHAP value slightly above 0.1. Interestingly, 3LFT, which performed well in linear regression, was ranked only fourth in importance. Generally, higher SPAD values contributed positively to LNC predictions, while lower SPAD values had a negative impact, except for 3LFT, which exhibited the opposite pattern.

When SPAD indices were used as input variables (**Figure 5B**), the difference SPAD indices (D2_1, D3_2, D4_2, D4_1) were the most influential for LNC prediction, followed by ratio and normalized difference indices. Adding statistical metrics as input variables (comb_3) significantly enhanced the model’s predictive power, increasing the R² from 0.44 to 0.73. In this combination, the top five most important features were statistical metrics (**Figure 5C**), with the median, maximum, and minimum values of 2LFT ranking highest. When using all features (**Figure 5D**), the median, original measurement, and maximum values of 2LFT remained the most influential, with average SHAP values far exceeding those of other features. Across all four feature combinations, the SPAD values of 2LFT and their derived indices consistently emerged as the most important variables, underscoring the critical role of 2LFT in estimating rice LNC.

In the SHAP analyses of the comb_2, comb_3, and comb_4 combinations, variables such as D3_2, R3_2, and ND3_2 consistently demonstrated that smaller values contributed more positively to LNC, indicating that a larger SPAD difference between 2LFT and 3LFT had a more significant positive impact. However, these variables had relatively lower influence on LNC predictions, ranking 6th, 9th, and 10th in importance in comb_3, and 4th, 6th, and 7th in comb_4. Although D3_2 ranked 2nd in comb_2, this combination had lower overall explanatory power for LNC.

**Figure 6:**
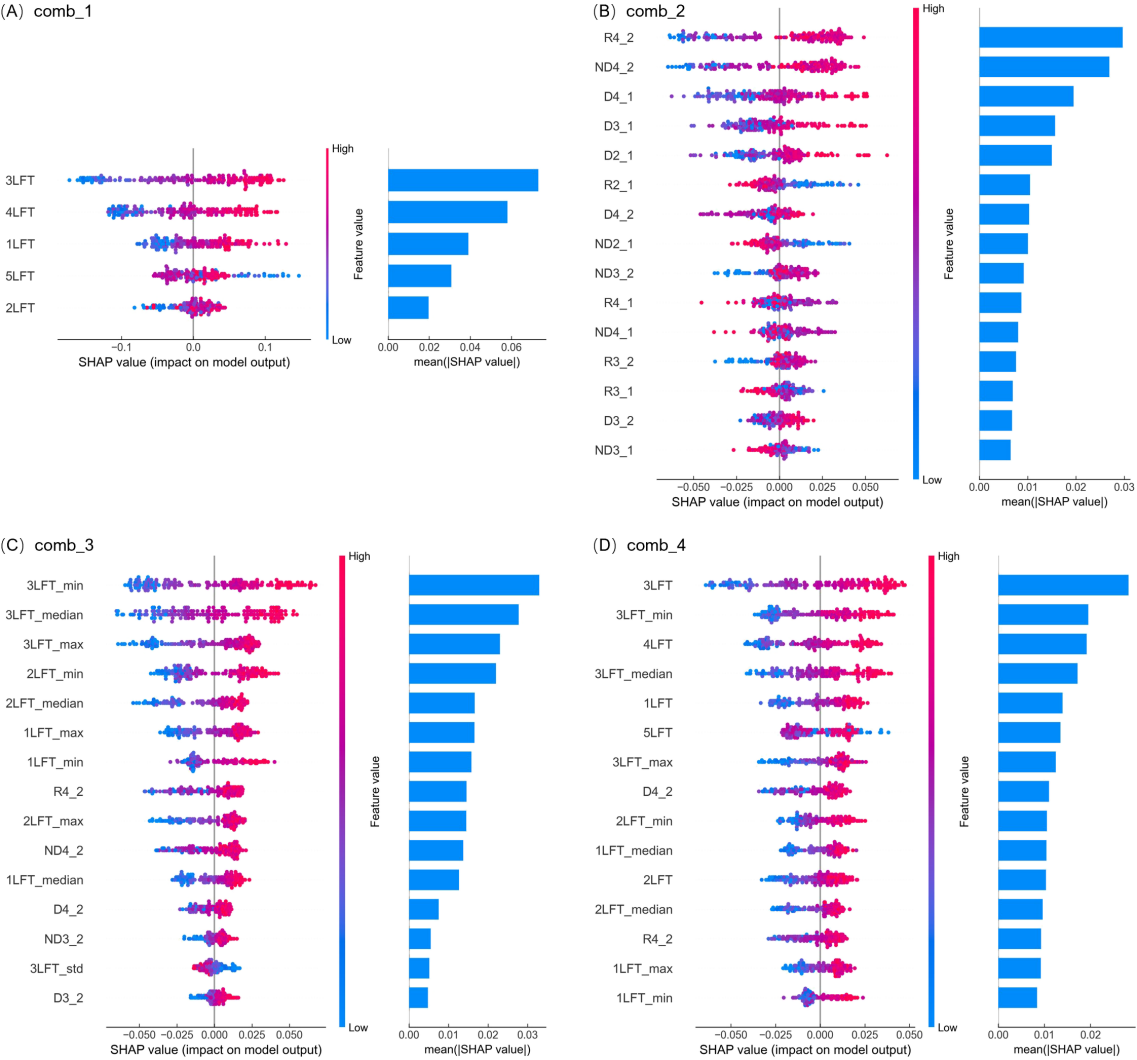
SHAP analysis of feature importance in predicting rice NNI with the random forest model across four input variable combinations. **(A–D)** correspond to the results for input variable combinations comb_1, comb_2, comb_3, and comb_4, respectively. The left side of each subplot shows SHAP value distributions, where each dot represents a data point from the validation dataset. The x-axis indicates the feature's impact on the model's prediction, and the dot color reflects the feature value, ranging from blue (low) to pink (high). Positive SHAP values indicate that the feature increases the prediction, while negative values indicate a decrease. The right side presents a bar chart (which shares the Y-axis with the left-side chart), with features ranked from top to bottom in descending order of their average absolute SHAP values, highlighting the most influential variables for the model's accuracy.

For NNI estimation, SHAP analysis revealed that when using only SPAD values as inputs (comb_1), 3LFT, 4LFT, and 1LFT were the most influential features, with 3LFT having the highest average SHAP value (**Figure 6**). The SHAP value distributions indicated that higher SPAD values at these leaf positions positively contributed to NNI predictions, suggesting better nitrogen status in the plant. When using SPAD indices as input variables (comb_2), R4_2, ND4_2, and D4_1 emerged as the most critical features, with larger values leading to higher NNI predictions. This pattern suggests that older leaves with higher SPAD values reflect a plant in a well-supplied nitrogen state.

When statistical metrics (such as median, minimum, maximum, and standard deviation) were added to the input variables (comb_3), the most important features were the minimum, median, and maximum SPAD values of 3LFT. Higher values of these metrics corresponded to higher NNI predictions, emphasizing that the distribution characteristics of SPAD measurements within a plot are key indicators of plant nitrogen status. When using all variables (comb_4), the model’s reliance on 3LFT SPAD values (3LFT, 3LFT_min, and 3LFT_median) remained strong, followed by 4LFT, consistent with LR results.

Additional validation results for LNC and NNI using the XGB model, along with SHAP analyses of feature importance, are provided in the **Supplementary Materials** (**Supplementary Figures S1**-**S3**). These results offer further insights into the model’s performance and the key variables influencing nitrogen estimation in rice.

## 4 Discussion

### 4.1 The relationship between rice leaf characteristics and plant nitrogen nutrition

The physiological state of rice leaves varies significantly depending on their position on the plant and their developmental stage (Yuan et al., 2016a; Sun et al., 2018). These variations critically influence leaf SPAD values and their relationship with LNC and the NNI. Younger leaves, particularly those at the top of the plant, are the primary growth centers and therefore receive prioritized resource allocation. These leaves typically undergo rapid growth, with quick expansion of leaf area and substantial increases in nitrogen and chlorophyll content. However, this dynamic growth phase results in greater measurement variability for the SPAD values of the uppermost leaves (Yuan et al., 2016b). Consequently, the correlation between SPAD values of the 1LFT and the plant’s overall nitrogen status tends to be lower compared to other leaf positions (**Tables 5**, **6**), consistent with findings from previous studies (Hu et al., 2014; Wang et al., 2014).

In contrast, the 2LFT and 3LFT are typically in a more stable phase of growth and physiological activity. These leaves are crucial for photosynthesis and nutrient assimilation, as they maintain relatively high levels of chlorophyll and nitrogen concentration. Research on different leaf layers within the rice canopy has shown that the nitrogen concentration in the second layer from the top (corresponding to 2LFT and 3LFT) is more stable compared to the lower and uppermost layers (He et al., 2022). This stability closely reflects the canopy-level nitrogen status, making it a crucial indicator for assessing the plant’s nitrogen status.

Meanwhile, the 4LFT and 5LFT typically enter or are already in the senescence phase, characterized by a decline in chlorophyll content, nitrogen concentration, and leaf area compared to 2LFT and 3LFT. This process aligns with the physiological redistribution of nutrients, wherein nutrients, including nitrogen, are translocated from older leaves to younger, actively growing parts of the plant. Under nitrogen-deficient conditions, this nutrient translocation occurs earlier and more prominently, leading to a marked decrease in SPAD values and skewed distribution patterns. Although SPAD values for 4LFT and 5LFT are more variable than those for 2LFT and 3LFT, the chlorosis and nutrient translocation observed in these leaves provide valuable insights into the plant’s overall nutritional status, making them useful indicators for diagnosing nitrogen nutrition in rice (Li et al., 2022b).

### 4.2 The role of multi-leaf variables and machine learning models in estimating plant nitrogen

Growth stages, rice cultivars, and environmental factors can significantly influence the accuracy of nitrogen nutrition diagnosis in crops (Huang et al., 2019; He et al., 2022; Lu et al., 2022). Delloye et al. (2018) reported that the relationship between canopy chlorophyll content retrieved from Sentinel-2 and actual nitrogen absorption in wheat was influenced by canopy structure complexity and saturation effects at different growth stages. One of the challenges in using SPAD measurements for nitrogen estimation is the saturation effect at high chlorophyll and nitrogen levels, where SPAD values plateau and become less sensitive to increases in nitrogen concentration (Gabriel et al., 2017; Souza et al., 2019). This saturation effect can reduce the sensitivity and accuracy of nitrogen diagnosis, particularly under high nitrogen availability.

In this study, the explanatory power of the linear regression between SPAD values and LNC and NNI significantly decreased after data consolidation, indicating substantial variability in these relationships across different growth stages and rice cultivars. This variability suggests that SPAD values from a single leaf position are insufficient for accurately predicting LNC or NNI. However, the SPAD values from 1-5LFT exhibit certain patterns of variation across different growth stages and nitrogen levels. By leveraging information from multiple leaf positions, especially those less prone to saturation, and incorporating features that reflect variability (e.g., minimum and median SPAD values), the models mitigate the impact of saturation on prediction accuracy.

Several studies have also demonstrated the contribution of multi-leaf position data to enhancing the accuracy of crop nitrogen nutrition diagnosis models (Lin et al., 2010; Zhao et al., 2018; Zhang et al., 2019). The use of Dualex (a leaf-clip meter that measures chlorophyll and flavonoid content) measurements from multiple leaf positions, combined with key environmental and management variables in multiple linear regression models, was critical in enhancing the precision of maize NNI estimation (Dong et al., 2021). By employing the Normalized SPAD Index (NSI), especially NSI4, Yuan et al. (2016a) achieved a notable improvement in the accuracy of nitrogen accumulation estimation, effectively reducing the influence of non-nitrogen-related factors.

Traditional statistical methods often rely on single or a limited number of variables, which can overlook the complex, nonlinear relationships that exist within biological data. In contrast, machine learning techniques offer significant advantages in managing multivariate inputs, allowing for the identification of intricate patterns and interactions (Chlingaryan et al., 2018). In our study, machine learning models like Random Forest (RF) and Extreme Gradient Boosting (XGB) demonstrated robustness in handling the nonlinear relationships caused by saturation effects. These models capture complex patterns by considering interactions among multiple features, including SPAD values from different leaf positions and statistical metrics. By leveraging data from leaves less affected by saturation and incorporating features that reflect variability, the models enhance the estimation of nitrogen status even when individual SPAD measurements reach their upper limits.

Among the four variable combinations tested, comb_4 consistently demonstrated superior performance in both LNC and NNI predictions. The superior performance of comb_4 can be attributed to its ability to leverage both raw SPAD values and derived indices. The inclusion of statistical metrics enhances the model’s robustness by accounting for variability and extremities in the data, which are critical for accurately assessing nitrogen status. Previous studies have highlighted the importance of using comprehensive feature sets in machine learning models, particularly in agricultural applications where complex biological processes often underlie the observed data (Lobell et al., 2015; Shi et al., 2021; Yang et al., 2023; Mandal et al., 2024). The success of comb_4 in this study aligns with these findings, underscoring the importance of detailed feature engineering in developing accurate predictive models for nitrogen estimation.

Previous research on diagnosing nitrogen nutrition in rice using leaf SPAD values has predominantly focused on normalized, ratio, or difference indices of SPAD, often achieving satisfactory estimation results (Lin et al., 2010; Zhao et al., 2018; Dong et al., 2021). These methods have been effective when applied to models built for single cultivars and specific growth stages, where SPAD values typically exhibit consistent patterns in relation to nitrogen supply. However, this study’s findings suggest that when using variable combination comb_2, which includes normalized difference indices, ratios, and differences between major leaf positions, the prediction accuracy was notably the lowest among the four tested combinations. This unexpected result may be attributed to the broader scope of this study, which encompassed multiple cultivars and growth stages, introducing additional complexity and variability that was not accounted for in previous studies. Under multi-cultivar and multi-growth-stage conditions, the generally narrow range of SPAD indices may fail to adequately capture the distinct nitrogen characteristics associated with different phenological and environmental factors. The variability introduced by these factors likely disrupts the otherwise consistent relationship between SPAD indices and nitrogen levels (Yuan et al., 2016a). As a result, the model using comb_2, which relies heavily on SPAD indices, may struggle to generalize across diverse datasets, leading to reduced prediction accuracy.

In contrast, variable combination comb_3 demonstrated significantly improved prediction accuracy. Unlike comb_2, comb_3 incorporated a broader range of features, including minimum, maximum, and median SPAD values. These features not only expand the range of input variables but also introduce statistical information that captures the variability and distribution of SPAD measurements in certain leaf positions. By doing so, comb_3 provides a more comprehensive representation of nitrogen status, allowing the model to more effectively distinguish between different growth stages and cultivars. This approach enhances the model’s robustness and generalization capability, resulting in better performance across a wide range of conditions.

### 4.3 Feature importance analysis in random forest models

The feature importance analysis in this study highlights the pivotal roles of the 2LFT and the 3LFT in estimating LNC and NNI, respectively. The findings indicate that higher SPAD values in 2LFT and 3LFT contribute significantly to increases in LNC and NNI, underscoring these leaf positions as key indicators of the plant’s nitrogen status.

This conclusion is supported by data from 17 independent experiments involving 15 rice cultivars, which consistently showed that 2LFT and 3LFT had the highest average SPAD values, with 2LFT slightly higher than 3LFT. The prominence of 2LFT can be attributed to its critical role in nitrogen allocation during the vegetative growth stage. As one of the younger, actively growing leaves, 2LFT receives a larger proportion of the plant’s nitrogen resources, which are essential for photosynthesis and biomass accumulation (Li et al., 2017; Wang et al., 2023). The relatively higher nitrogen concentration and larger leaf area of 2LFT increase its overall nitrogen content, making it a more accurate representative of the plant’s total leaf nitrogen status.

In contrast, 3LFT, which transitions from maturity to senescence, is identified as the most critical leaf position for estimating NNI. Previous research has shown that leaf development and senescence are closely linked to nitrogen availability, with low soil nitrogen supply accelerating leaf chlorosis and senescence (Heyneke et al., 2019; Li et al., 2022b). As 3LFT moves from maturity to senescence, its SPAD value becomes a crucial marker of the plant’s nitrogen status, especially in the early stages of vegetative growth when the number of leaves is limited. This makes 3LFT a valuable indicator for assessing plant NNI, particularly under conditions of nitrogen deficiency, where early onset of senescence in this leaf position can signal broader nutritional challenges within the plant (Sun et al., 2018).

While multi-leaf SPAD measurements enhance estimation accuracy, we recognize that collecting data from multiple leaves may not be feasible for all farmers, particularly those with limited resources or on smaller farms. To balance complexity and practicality, focusing on the most critical leaf positions identified by our feature importance analysis offers a viable solution. By concentrating on SPAD measurements from 2LFT for LNC prediction and 3LFT for NNI estimation, data collection can be simplified without substantially compromising accuracy. Implementing this targeted approach can make the method more accessible and practical for widespread adoption, facilitating efficient nitrogen management in rice cultivation. Future studies should explore the development of user-friendly tools or protocols that assist farmers in easily collecting SPAD data from these specific leaf positions.

## 5 Conclusion

This study demonstrates the effectiveness of using multi-leaf SPAD values and advanced machine learning models—particularly RF and XGB—to accurately estimate LNC and the NNI in rice. The 2LFT consistently emerged as the most critical variable for LNC prediction, while the 3LFT was pivotal for NNI estimation. Incorporating statistical metrics, such as maximum and median SPAD values, significantly enhanced model performance, underscoring the importance of considering both original SPAD measurements and derived indices.

While multi-leaf SPAD measurements improve estimation accuracy, focusing on the key leaves identified by our analysis can simplify data collection, making the method more practical for farmers. Targeted monitoring of SPAD values in these specific leaf positions can improve the precision of nitrogen assessments, thereby enhancing crop management practices and optimizing nitrogen use efficiency for sustainable agriculture. Future research should focus on refining these models under varying environmental conditions, exploring their applicability to other crops, and addressing challenges related to model generalizability and integration with other data sources.

## Data Availability

The original contributions presented in the study are included in the article/**Supplementary Material**. Further inquiries can be directed to the corresponding author.
