# Supplementary material for: Enhancing Nitrogen Nutrition Index estimation in rice using multi-leaf SPAD values and machine learning approaches
Source: Front Plant Sci. 2024 Dec 10;15:1492528. doi: 10.3389/fpls.2024.1492528 (PMC11666358; doi:10.3389/fpls.2024.1492528)
Supplement: Supplementary file 1 [file DataSheet1.docx]

Supplementary Material

# Supplementary Figures


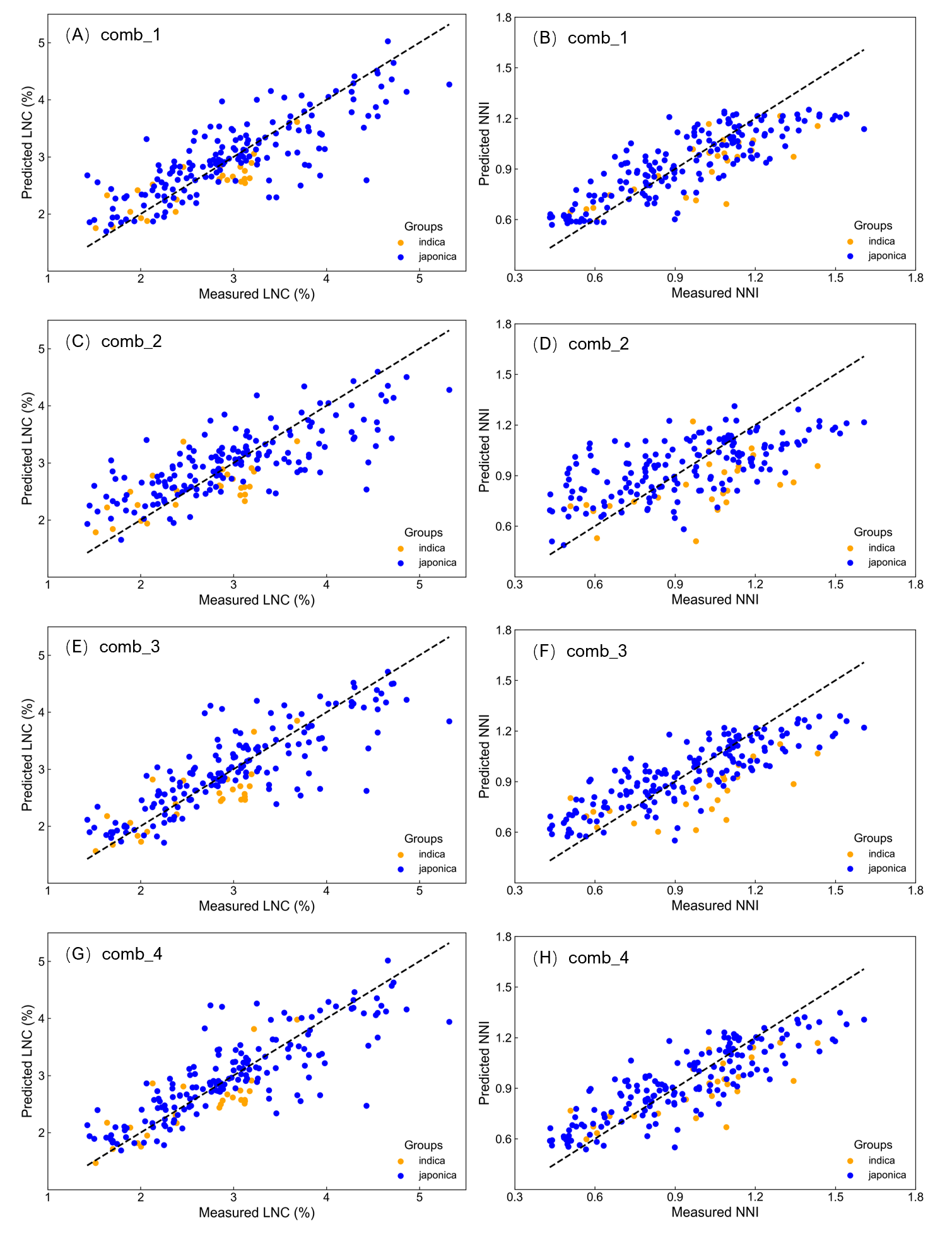


**Supplementary Figure 1.** Validation results for predicted vs. measured LNC (A, C, E, G) and NNI (B, D, F, H) using the extreme gradient boosting with input variable combinations comb_1, comb_2, comb_3, and comb_4, respectively. Each plot shows 1:1 scatter relationships.


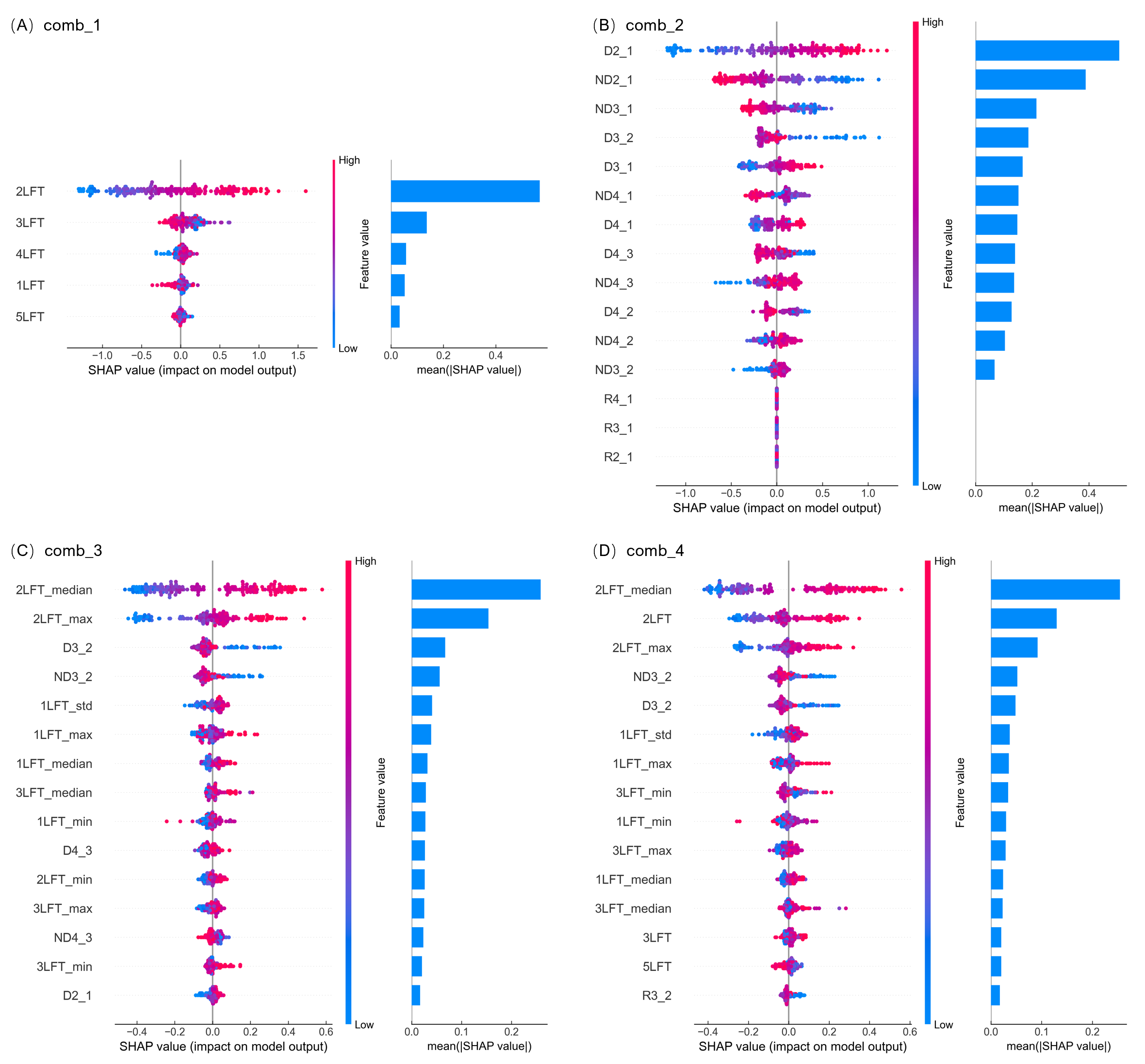


**Supplementary Figure 2.** SHAP analysis of feature importance in predicting LNC with the extreme gradient boosting model across four input variable combinations. (A, B, C, D) correspond to the results for input variable combinations comb_1, comb_2, comb_3, and comb_4, respectively. The left side of each subplot shows SHAP value distributions, where each dot represents a data point from the validation dataset. The x-axis indicates the feature's impact on the model's prediction, and the dot color reflects the feature value, ranging from blue (low) to pink (high). Positive SHAP values indicate that the feature increases the prediction, while negative values indicate a decrease. The right side presents a bar chart (which shares the Y-axis with the left-side chart), with features ranked from top to bottom in descending order of their average absolute SHAP values, highlighting the most influential variables for the model's accuracy.


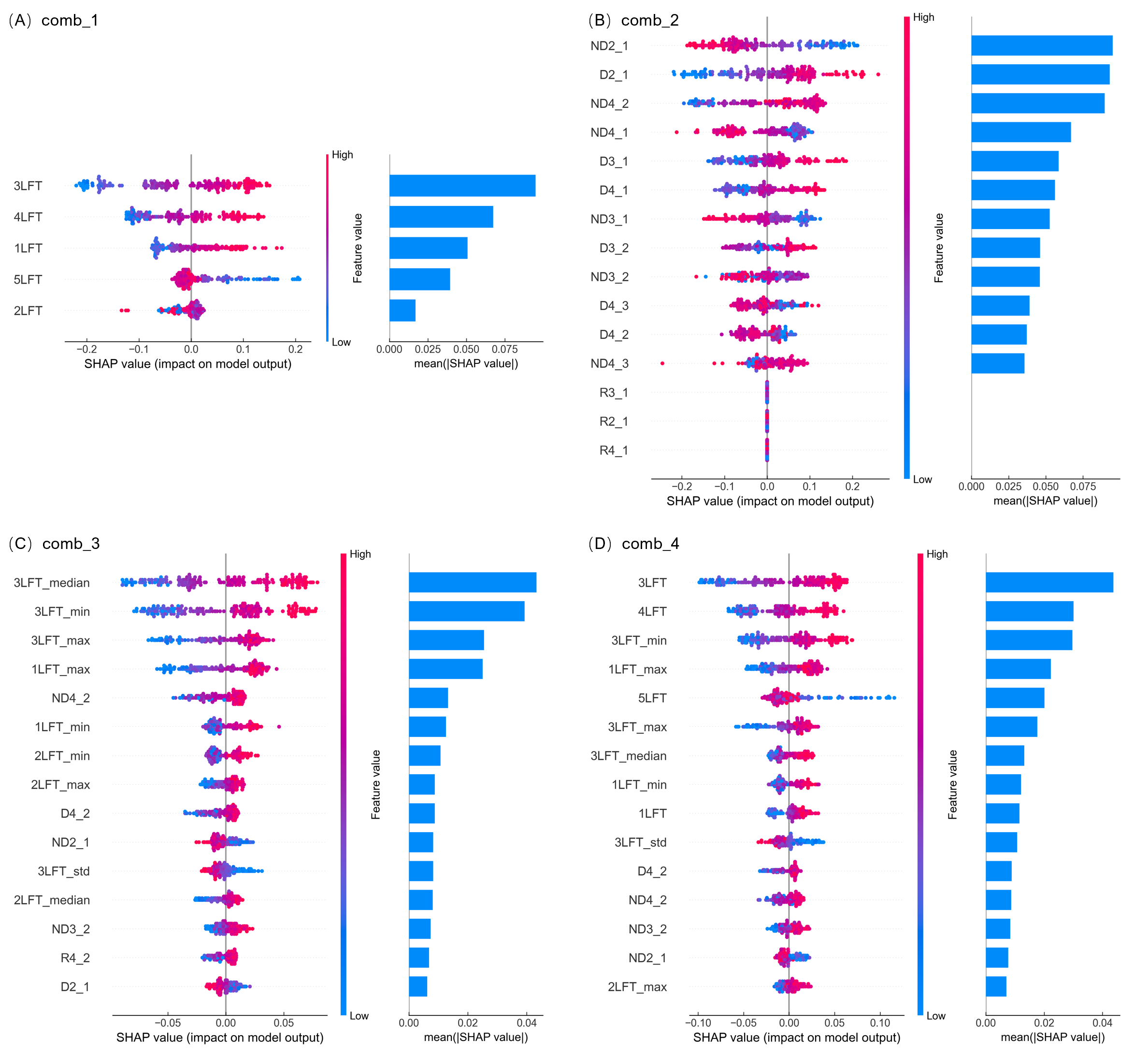


**Supplementary Figure 3.** SHAP analysis of feature importance in predicting rice NNI with the extreme gradient boosting model across four input variable combinations. The left side of each subplot shows SHAP value distributions, where each dot represents a data point from the validation dataset. The x-axis indicates the feature's impact on the model's prediction, and the dot color reflects the feature value, ranging from blue (low) to pink (high). Positive SHAP values indicate that the feature increases the prediction, while negative values indicate a decrease. The right side presents a bar chart (which shares the Y-axis with the left-side chart), with features ranked from top to bottom in descending order of their average absolute SHAP values, highlighting the most influential variables for the model's accuracy.
